# Performance Evaluation and Comparison between Direct and Chemical-Assisted Picosecond Laser Micro-Trepanning of Single Crystalline Silicon

**DOI:** 10.3390/ma12010041

**Published:** 2018-12-23

**Authors:** Hao Zhu, Zhaoyang Zhang, Kun Xu, Jinlei Xu, Shuaijie Zhu, Anbin Wang, Huan Qi

**Affiliations:** 1 School of Mechanical Engineering, Jiangsu University, Zhenjiang 212013, China; haozhu@ujs.edu.cn (H.Z.); xukun@ujs.edu.cn (K.X.); 18852867426@163.com (J.X.); shuaijiezhu@163.com (S.Z.); 17364399730@163.com (A.W.); 2 Key Laboratory of Special Purpose Equipment and Advanced Processing Technology, Ministry of Education & Zhejiang Province, Zhejiang University of Technology, Hangzhou 310014, China; huanqi@zjut.edu.cn

**Keywords:** micro-trepanning, single crystalline silicon, picosecond laser, chemical-assisted, laser-material interaction, material removal mechanism

## Abstract

The fabrication of micro-holes in silicon substrates that have a proper taper, higher depth-to-diameter ratio, and better surface quality has been attracting intense interest for a long time due to its importance in the semiconductor and MEMS (Micro-Electro-Mechanical System) industry. In this paper, an experimental investigation of the machining performance of the direct and chemical-assisted picosecond laser trepanning of single crystalline silicon is conducted, with a view to assess the two machining methods. The relevant parameters affecting the trepanning process are considered, employing the orthogonal experimental design scheme. It is found that the direct laser trepanning results are associated with evident thermal defects, while the chemical-assisted method is capable of machining micro-holes with negligible thermal damage. Range analysis is then carried out, and the effects of the processing parameters on the hole characteristics are amply discussed to obtain the recommended parameters. Finally, the material removal mechanisms that are involved in the two machining methods are adequately analyzed. For the chemical-assisted trepanning case, the enhanced material removal rate may be attributed to the serious mechanical effects caused by the liquid-confined plasma and cavitation bubbles, and the chemical etching effect provided by NaOH solution.

## 1. Introduction

The importance of micro through-holes can be represented by many applications, from solar cell and micro-devices to injection nozzles and turbine blades [1,2,3,4,5]. Taking the solar cell as an example, to avoid the shading effect of front electrodes, dense via holes that have a higher aspect ratio and small diameter are fabricated in solar panels to realize emitter wrap-through (EWT) or metal wrap-through (MWT) design, so that conventional top contact with front electrodes can be shifted to the rear through via holes, which increases the energy conversion efficiency [5]. For turbine blades working under a high-temperature flame, small holes are usually drilled to form an effusion cooling system, so that the thermal stress that is applied on the blade surface can be reduced, and premature failure can be avoided [3].

Laser technology has been extensively used in material micro-processing fields due to its ability to focus energy into a small irradiation area within a short pulse duration, and hence lead to material removal, regardless of the mechanical properties of the material [6,7]. In terms of micro-drilling application, laser has shown good performance, and several drilling techniques have been proposed according to the relative movement between the laser beam and the workpiece, such as percussion drilling [2], trepanning [8], and laser helical drilling [9]. Among these drilling schemes, laser trepanning appears to be more competitive due to its flexibility with laser scanning path control, enabling it to make free-form holes with different contours at the hole entrance/exit [3,8]. Therefore, the laser trepanning scheme has been widely employed for micro-hole production in various materials, such as superalloy [3,8], titanium alloy [10], tungsten carbide [4], polymers [11], and ceramics [12].

The micro-machining of semiconductor materials, which is typified by silicon, has been attracting intense interest for a long period due to its importance in industry application. Drilling micro-holes in silicon substrates with proper taper, a higher depth-to-diameter ratio, and better surface quality is a lasting goal along with the fast development of the semiconductor industry, and many researchers are focusing on this field. Lee and Choi [2] carried out silicon via hole-drilling analysis using a ultraviolet (UV) nanosecond (ns) laser, pointing out that the via hole depth shows a linear relationship to the pulse number and a logarithmic relationship with the laser fluence. Tan [1] also chose a ns UV laser for micro-hole drilling in silicon substrate, and found that the shape deformation of the machined hole can be attributed to the high pressure caused by the expanding vapor/plasma and the recoil of the ablated material. In a ns laser helical drilling of silicon completed by Kaspar et al. [9], thick recast layers were observed deposited on the sidewall that could not be fully avoided through parameter optimization, indicating that the ns laser is not qualified for the high precision and low damage requirements. Brandi et al. [13] explored the effect of laser spot size on the ns laser percussion drilling efficiency of silicon, and found that the ablation efficiency increases up to 10 times when the spot diameter is decreased from 220 to 90 μm at a given fluence range, while the debris and resolidification phenomena around the hole edge are still obvious in the drilling results.

Compared with the ns laser, the ultrashort pulsed laser is associated with a different laser–material interaction mechanism, enabling it to modify material property directly [14] or fabricate a micro-device accurately [15]. In terms of the micro-drilling of silicon, the ultrashort pulse laser has also demonstrated its capability in minimizing thermal damage. Kaspar et al. [9] compared the laser drilling performance when pulse duration varied from ns to femtosecond (fs), i.e., eight ns, 350 ps, 10 ps, one ps, and 160 fs, pointing out that the smooth holes that are recast layer-free without thermal and mechanical damage can be achieved with a pulse width of 10 ps. In contrast, ns laser drilling suffers from microcrack formation, high thermal load, and obvious melt redeposition, while fs laser drilling results are associated with mechanical defects, including the microroughness of the sidewall and material degradation. Ahn et al. [5] conducted fs laser drilling with crystalline and multicrystalline silicon as targets, and two distinct material ablation mechanisms were found: one governed by the direct incident laser beam at the early stage, and the other dominated by the forming hole wall-guided beam at the subsequent stage. In addition, the crystalline silicon was found to be associated with a lower material removal rate compared with the multicrystalline one at the latter stage, which can be attributed to the stronger intrinsic structure and higher absorption coefficient of crystalline silicon. Jiao et al. [16] investigated the effect of silicon substrate temperature on the fs laser drilling process with respect to hole quality and drilling efficiency, and revealed that an increase in substrate temperature within a range of 300 to 873 K leads to a rapid increase in laser drilling efficiency and a decrease in the spatter area surrounding the hole edge, which may be attributed to the temperature-enhanced absorption coefficient of silicon to the laser energy. Coyne et al. [17] stated that although the fs laser machining of silicon can effectively minimize thermal damage, the ablated material deposition and mechanical stresses that cause lattice defects still have negative effects on the machining results.

Apart from direct laser machining, liquid has been introduced into the laser drilling process as a thin layer to effectively remove ablated debris and improve hole quality [18,19,20]. Kaakkunen et al. [21] carried out water-assisted fs laser drilling of silicon, and concluded that an obvious increase in the machining efficiency and hole quality can be achieved with help of a thin water layer compared with that in air atmosphere, which can be attributed to the reduced incident laser energy loss due to the immediate removal of ablation debris by the flowing water. A similar study has been conducted by Wee et al. [20], in which the ns laser trepanning performances of silicon wafer in air and under water were compared. It was pointed out that the flowing water film behaving as a cooling media effectively prevents spatters deposition and results in a decreased hole taper. Meanwhile, laser trepanning in air was found to be associated with a smoother ablated surface compared with that achieved by underwater machining. Jiao et al. [18] carried out a volatile liquid-assisted fs laser drilling study of silicon by introducing a thin liquid layer with a thickness estimated around 0.1–0.2 mm, concluding that a more volatile liquid with a lower boiling point and less carbon atoms in the molecule formula is more effective in eliminating laser-caused debris, which may be mainly attributed to the liquid-confined shock wave, which varies with the surrounding liquids.

To further enhance the machining efficiency and improve the ablated surface quality, many researchers have introduced chemical reactions into the laser drilling process. The gaseous environments of certain chemical materials such as sulfur hexafluoride (SF_6_), hydrogen chloride (HCl), and chlorine (Cl_2_) have been created surrounding the target materials, and a higher laser processing efficiency for silicon and germanium can be achieved, which is due to laser ablation and laser-assisted chemical etching [22,23,24]. Apart from gaseous atmosphere, chemical material can also be introduced as a liquid, and a chemical reaction may be activated or accelerated by the laser-caused high temperature near the irradiating area [25], leading to an increased material removal rate and better surface quality. Hopman et al. [26] employed potassium hydroxide (KOH) solution in the liquid jet-guided laser machining of silicon, and found that the debris problem that is typically associated with laser machining can be reduced compared with water conditions, although the full elimination of debris has not been achieved. In a similar comparative study carried out by Baumann et al. [27], the amorphous silicon that is generated during laser processing may be effectively removed by the KOH solution, so that the crystallinity of silicon is retained after processing. Akhter et al. [28] pointed out that the redox reaction between silicon and sodium hydroxide (NaOH) solution is highly temperature-dependent, and a higher solution temperature leads to increased etching efficiency. In addition, the etched surface becomes considerably smoother with an increase in NaOH solution concentration or temperature, leading to improved surface quality.

In this study, an orthogonal experimental exploration of direct and chemical-assisted laser trepanning of silicon is carried out, and the machining results are compared in terms of surface quality and hole geometry. Range analysis is then conducted to evaluate the influences of processing parameters on the trepanning results, and recommendations regarding the trepanning method and parameters combination are also given. Finally, the material removal mechanisms that are associated with the two trepanning methods are amply discussed.

## 2. Materials and Methods

### 2.1. Experimental Set-Up

**Figure 1:**
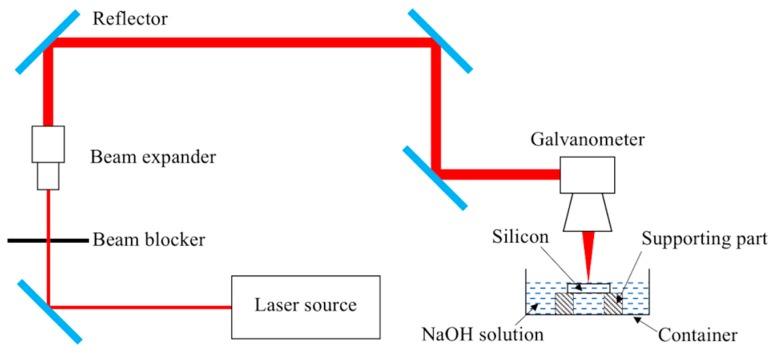
Schematic of the picosecond laser micro-trepanning system.

The optical schematic of a laser drilling system (Delphi Laser Co. Ltd., Suzhou, China) is shown in Figure 1, in which a Nd:YVO_4_ laser (Edgewave PX100-1-GM, EdgeWave GmbH, Aachen, Germany) operating in Gaussian mode was included. The emitted laser beam runs with a ~12 ps pulse width at a 1064 nm wavelength, while the output power is up to 70 W. The laser pulse frequency is adjustable between 0.2–1 MHz, in which range the output power stays nearly constant, leading to a maximum single pulse energy of around 260 μJ at a 0.2 MHz pulse frequency. For this ps laser machine, the high voltage (HV) level working on the HV modulator was employed to adjust the output power, and this power adjustment scheme has an advantage over the pump current control method, as the varied HV level does not affect the laser spot size. In the optical path, a beam expander (Eoptics VE-532-1064, JENOPTIK, Jena, Germany) was used to enlarge the emitted laser beam, and the expanded beam was then directed to a galvanometer (IntelliSCAN 14-1064, SCANLAB, Munchen, Germany) with a focal length of 100 mm and a typical marking velocity of 2 m/s, after which the laser beam finally reached the target surface to induce a laser-material interaction. For a safety protection purpose, a mechanical beam blocker was added in the optical path. To ensure that the laser machine operates stably, filtered and deionized water was looped inside it to maintain a constant working temperature. Using this optical set-up, the focused laser spot in the focal plane was around 20 μm in diameter.

Both direct and chemical-assisted laser trepanning of silicon are carried out in this study. For direct laser dry machining, the container shown in Figure 1 is empty, while in the chemical-assisted trepanning case, the container is filled with a proper amount of NaOH solution to ensure that the layer thickness above the silicon surface is around one mm. A large container is chosen to minimize the fluctuation of the solution height caused by the laser-induced shockwave or evaporation during the trepanning process.

**Figure 2:**
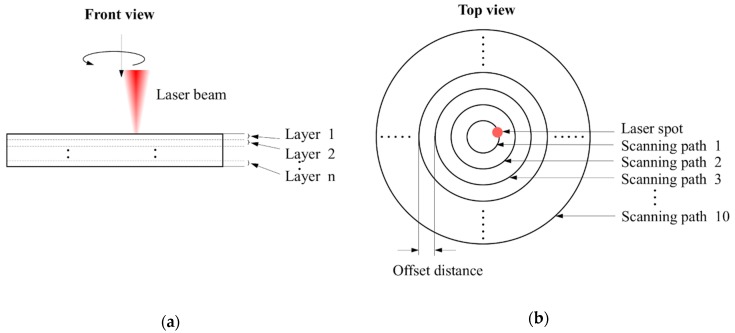
Schematic diagram of laser trepanning, where (**a**) represents the front view, while (**b**) corresponds to top view.

In this study, the target specimens were single crystalline silicon plates with a thickness of about 0.6 mm, and the main physical and mechanical properties are listed in Table 1. The target through hole was fabricated following the trepanning scheme illustrated in Figure 2, where the material removal was realized layer by layer in a vertical direction, i.e., from layer one to layer *n*, as the focused plane position (*fpp*) of the laser beam feeds downwards regularly. The feeding step (*d*) between two adjacent layers was calculated as:

*d* = *L*/*n*, (1)

where *n* is the layer number, and *L* theoretically represents the specimen thickness, but was set a little larger practically, i.e., 0.7 mm in this study, to make sure that the accumulated feeding distance of the focal position surpasses the substrate thickness. At each layer, the laser spot circles along concentric scanning paths from one to 10 with a constant offset distance of 10 μm, corresponding to diameters from 20 to 200 μm, respectively. Laser scanning repeats followed these paths for certain times at each layer, which were named element number (*m*). Therefore, the final hole formation was the accumulated material removal results corresponding to each laser heating path at each layer.

### 2.2. Experiment Design

In this laser trepanning process, the laser is utilized to heat the specimen and may lead to melting, vaporization, plasma generation, or direct phase explosion as in the fs laser processing condition. In addition, the chemical reaction between silicon and NaOH solution also enhances material removal. As a result, the factors that affect laser heating and the chemical reaction process may influence the material removal and hence hole formation. However, compared with the ultrafast laser ablation process, chemical etching is usually associated with a lower efficiency [28], so its effect on material removal is comparatively less significant considering the short processing duration here. Therefore, only laser-related parameters were focused on and explored in this experimental study, where the laser power (*P*) representing the measured output power arriving at the target surface, laser pulse frequency (*f*), laser beam scanning velocity (*v*), layer number (*n*), and element number (*m*) were chosen to assess the trepanning process, while the concentration of NaOH solution was kept at ~1 mol/L. The testing levels of the selected laser parameters were determined based on their available adjusting range in the laser trepanning system and the preliminary experimental results, as listed in Table 2. Four levels were chosen for each parameter, and the orthogonal design method was used so that the experimental test had a manageable size. An L_16_(4^5^) orthogonal array with 16 rows and five columns was employed, resulting in 16 sets of parameter combinations. To reduce the uncontrollable influence, each combination of parameters was repeated five times, resulting in 80 holes for direct drilling and 80 for chemical-assisted machining, respectively.

**Figure 3:**
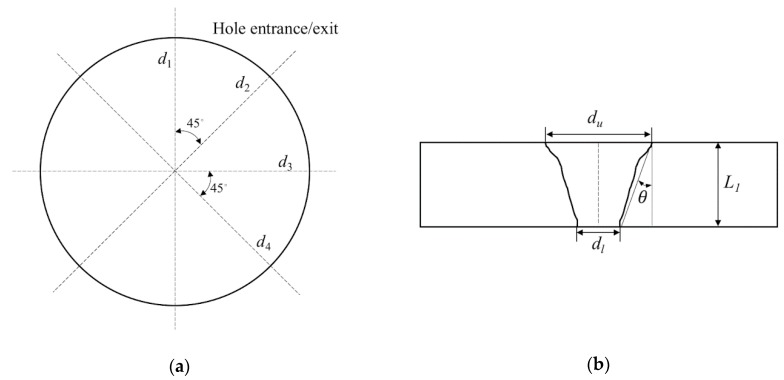
Schematics of (**a**) diameter measurement at the entrance and exit of the trepanning result, and (**b**) hole taper estimation.

Measurements of the hole diameters at the entrance and exit sides, as well as observations of the hole characteristics, were carried out after machining to assess the trepanning performance under different parameter combinations. The hole diameters were measured using the CCD microscope (the IMAGING SOURCE, Bremen, Germany) integrated in the advanced ps laser machining system, and four orientations with intervals of 45° were selected for each hole measurement, as illustrated in Figure 3a. In this way, each parameter combination resulted in 20 diameter readings both at the entrance and exit, and the average was accepted as the final reading. Afterwards, hole taper can be estimated as:

*θ* = arctan((*d_u_* − *d_l_*)/(2*L*_1_)), (2)

where *θ*, *d_u_*, *d_l_*, and *L*_1_ represent the hole taper, averaged entrance diameter, averaged exit diameter, and the actual silicon plate thickness, respectively. The hole characteristics were also observed under the scanning electron microscope (SEM, Hitachi S-3400N, Hitachi, Tokyo, Japan) for qualitative analysis.

## 3. Results

### 3.1. Comparison between Direct and Chemical-Assisted Laser Trepanning

**Figure 4:**
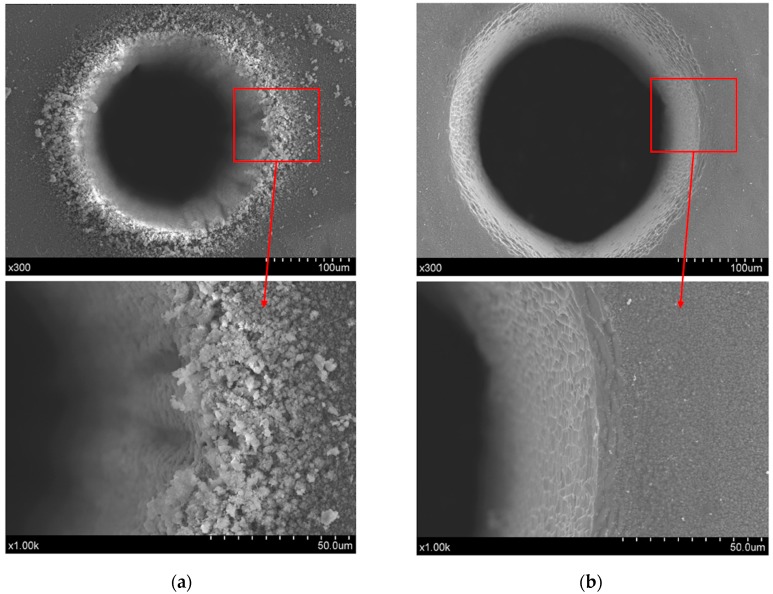
Entrance comparison of the micro-trepanning results, in which (**a**) corresponds to direct laser machining, while (**b**) represents the chemical-assisted method. The processing parameters include: *P* = 18.5 W, *f* = 0.4 MHz, *v* = 600 mm/s, *n* = 20, *m* = 120, and an NaOH solution with a concentration of one mol/L was employed in the chemical-assisted laser processing.

The comparison of trepanning results yielded by direct and chemical-assisted laser machining methods are carried out to evaluate their performances, and the surface characteristics around the hole entrance are shown in Figure 4, in which Figure 4a corresponds to direct laser trepanning, while Figure 4b represents the chemical-assisted method. It is clearly illustrated in Figure 4a that the laser dry trepanning result is associated with an obvious spatter area around the hole entrance, and such accumulated spatters may form a pile-up along the entrance edge. In addition, spatters can be noticed at positions nearly 100 μm away from the hole edge, suggesting that the material is removed in a severe “explosion” way, as no assisted gas is involved in the direct laser trepanning process. Furthermore, the spatters are quite even in size, most of which are within ~5 μm, as shown in the lower part of Figure 4a. For chemical-assisted laser trepanning results, a clean entrance surface with negligible spatters deposition can be observed in Figure 4b, in which the hole entrance edge is clear and sharp. Moreover, a much smoother sidewall is noticed in contrast to the rough one caused by direct laser trepanning that is shown in Figure 4a, which should be attributed to the atom-by-atom material removal manner during the chemical etching process, which may be further enhanced by high temperature [28].

It is also noted that the chemical-assisted laser trepanning is associated with a wider hole compared to the direct one under the same processing parameters, which may be mainly attributed to the laser-induced plasma and the following cavitation bubbles in the assisted liquid. For the ps pulsed laser employed in this study, the peak laser intensity is up to around 700 GW/cm^2^ [30], which is sufficient to trigger and maintain plasma in the assisted liquid [31]. Such a plasma zone exhibits an obvious shielding effect on the incident laser energy via laser beam reflection and energy absorption, causing laser energy loss along the beam propagation direction and a high temperature up to 10^4^–10^5^ K inside the plasma zone [32]. Therefore, a high-temperature plasma zone may further heat the substrate as another heat source besides the laser beam. Meanwhile, the dynamic plasma zone expands rapidly toward the surrounding media, and a more severe expelling effect can be produced in the assisted liquid than in air, leading to high-pressure of up to several GPa [33], and hence a strengthened plasma shock wave. After the laser pulse, the plasma zone decays fast and degenerates into cavitation bubbles, which then collapse and lead to liquid jet impact and shock waves on the substrate. Consequently, a serious mechanical effect is likely to be applied on the processing position, and the material removal is reasonably enhanced. Moreover, the chemical reaction rate between silicon and NaOH solution will also probably get accelerated due to the high local temperature generated by laser heating [28], resulting in a higher material removal rate. As a result, wider holes are expected in chemical-assisted trepanning.

**Figure 5:**
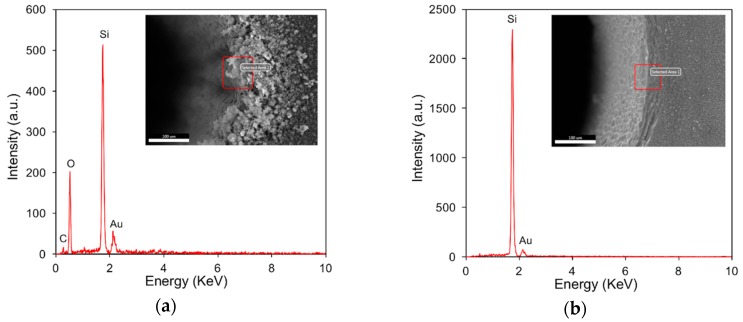
EDS (energy dispersive spectrometer) analysis results at given positions of the hole entrance edge marked by red squares, where (**a**) corresponds to direct laser machining, while (**b**) represents chemical-assisted trepanning. The processing parameters are same as those in Figure 4.

To examine the oxidation phenomenon that is typically associated with laser machining due to the thermal effect, EDS analyses were conducted at selected positions, i.e., the hole edge, and the EDS spectra are shown in Figure 5. Regarding the laser dry trepanning results, oxygen (O) has been detected with a non-negligible peak amplitude accounting for nearly half of the silicon, as illustrated in Figure 5a, demonstrating that laser-caused oxidation exists, and the spatter seems to be the oxide of silicon. In contrast, the peak corresponding to O has not been observed in the chemical-assisted laser trepanning case, as shown in Figure 5b, indicating that the ablated surface is free of oxidation after processing. Plus, aurum (Au) has been noticed in both trepanning conditions, which should be attributed to the specimen pretreatment before SEM, as a thin Au layer was deposited on the target surface to enhance the electrical conductivity for clearer observations.

**Figure 6:**
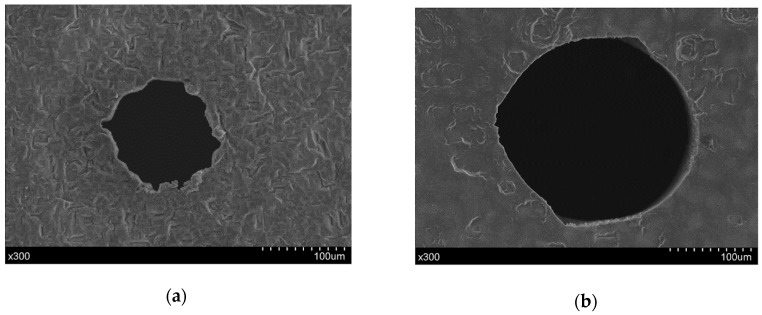
Exit comparison of the micro-trepanning results, in which (**a**) corresponds to direct laser machining, while (**b**) represents chemical-assisted trepanning. The processing parameters are the same as those in Figure 4.

A comparison of the hole exit characteristics between the direct and chemical-assisted laser trepanning results is shown in Figure 6, in which the processing parameters are the same as those in Figure 4 and Figure 5. Spatters around the exit edge can be observed in the direct trepanning case that is shown in Figure 6a, while the hole edge is comparatively clean and clear in the chemical-assisted trepanning condition, as illustrated in Figure 6b. Meanwhile, the chemical-assisted method results in an obviously wider exit than that in the direct method, which may also be attributed to the mechanical effects caused by liquid-confined plasma and cavitation bubbles, as well as temperature-enhanced chemical etching, as analyzed before. In addition, the chemical-assisted method is associated with better hole roundness at the exit, whereas the direct one results in an exit where the edge seems quite arbitrary at several locations, which further demonstrates the advantage of chemical-assisted laser trepanning over direct laser machining.

### 3.2. Effects of Parameters on Direct Laser Trepanning

The measured results of direct laser trepanning in this orthogonal experiment are summarized in Table 3, where the averaged readings of the entrance and exit diameters, as well as the hole taper, are listed. The standard deviations of the entrance and exit diameters are also given, as represented by σ-Entrance and σ-Exit, respectively. For the direct laser trepanning process, the material removal is completely attributed to the laser–material interactions, as no assisted gas was employed, so that the ablated material may be not removed immediately. Hence, redeposition may occur, especially in the limited transition hole space. In addition, the material removal efficiency usually decreases with an increase in hole depth [34]. As a result, a blind hole may be produced under some combinations of parameters rather than a through hole, as for tests L2, L3, L4, L7, and L12.

**Figure 7:**
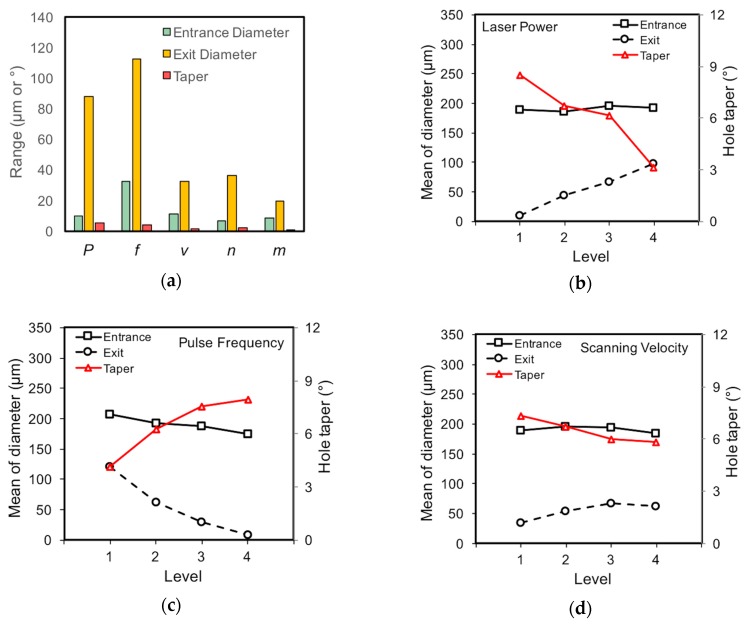

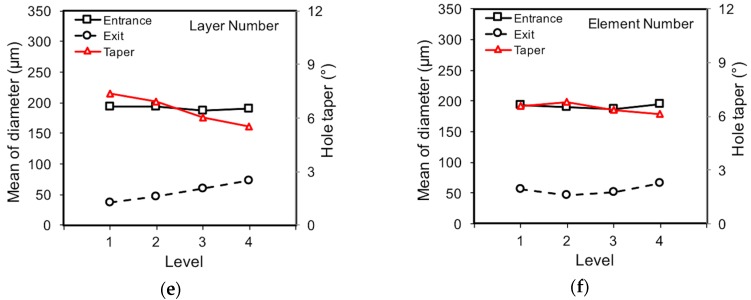
Effects of processing parameters on direct trepanning results, where (**a**) is the range analysis result, and (**b**–**f**) correspond to the laser power, pulse frequency, scanning velocity, layer number, and element number, respectively.

The measured results that are summarized in Table 3 have been visualized to clearly illustrate the effects of each parameter on the trepanning result, as shown in Figure 7. Range analysis is conducted following the calculation details described in previous work [30], and the range values of selected parameters with respect to the entrance/exit diameter and hole taper are shown in Figure 7a. Considering the entrance diameter, the laser pulse frequency has most significant effect, followed by the scanning velocity, while the laser power, layer number, and element number are associated with comparatively less significant effects. For the diameter at the exit side, the pulse frequency and laser power far surpass the other parameters affecting the significance, followed by the scanning velocity and layer number, while the element number has the smallest effect. When the hole taper is considered, the effects of the laser power and pulse frequency are noticeable, while the others are considerably insignificant.

In an orthogonal experiment, the orthogonal array is well designed so that the level of each factor varies evenly, and therefore, the mean of the measured results can be used to evaluate the influencing effects of certain factors on the machining results [30]. Figure 7b depicts the changing trends of the measured indicators, i.e., the entrance diameter, exit diameter, and hole taper, with respect to laser power. Specifically, the entrance diameter increases slightly with an increase in the laser power from 11.5 to 32 W (corresponding to levels one to four), while such an increase in laser power results in a more significant rise in exit diameter. As a result, the hole profile becomes increasingly steeper, and the hole taper shows a decreasing trend with respect to the laser power within the selected range. These changing trends may be interpreted from a view of laser-material interaction. Since laser heating repeats a certain number of times (the element number) at each layer along the laser scanning paths, as shown in Figure 2, the incident laser intensity in this study and the accumulated input energy at the top surface are high enough to surpass the ablation threshold, so that even the lowest laser power is able to sufficiently remove material, and a higher laser power only leads to a slight increase in the entrance diameter. In contrast, the material removal efficiency decreases obviously with an increase in the hole depth [34], which may be partially attributed to the laser energy loss when propagating and reaching the hole bottom. Such an energy loss may be owing to the shielding effect of the vapor/plasma that is formed by material evaporation/ionization, as well as the laser energy absorption and reflection by the sidewall or top surface during the downward feeding of the laser focal plane position. Therefore, the transmitted laser energy arriving at the bottom or lower sidewall of the transition hole to further cause material removal is reduced, and such transmitted laser energy decreases with an increase in the hole depth. According to the experimental results shown in Figure 7b, it appears that a higher input laser power leads to higher transmitted energy at the hole bottom, and hence more material removal and a wider exit are expected, as well as a smaller estimated hole taper. However, for the lowest laser power level, the mean exit diameter is likely to approach 0, suggesting that the transmitted energy is too small to pierce the workpiece, and hence a blind hole rather than a through one is produced.

The effects of laser pulse frequency on the trepanning results are shown in Figure 7c, in which an increase in pulse frequency from 0.4 to 1.0 MHz (level one to four) decreases both the entrance and exit diameters, while the latter is associated with a higher declining rate, thus leading to bigger hole tapers. For a given laser power, the single pulse energy decreases with an increase in pulse frequency, i.e., from 28.75 μJ at 0.4 MHz to 11.5 μJ at 1.0 MHz under the laser power of 11.5 W, so that the laser–material interaction weakens, and less material can be ablated per single pulse. Although the material ablation rate may be enhanced owing to the accumulated material removal effect caused by the increased pulse frequency, it appears that such a possible increase in the material removal efficiency does not trade off the decreasing trend due to the reduced material removal rate per single pulse. Therefore, both the entrance and exit diameters illustrate declining trends with the pulse frequency.

The effects of laser scanning velocity are illustrated in Figure 7d, in which the entrance diameter varies slightly within the selected parameter range. Again, this can be attributed to the incident laser intensity and accumulated input energy near the top surface being high enough for sufficient material removal, and thus, the laser-irradiated area roughly equals the ablation area, leading to slightly varied entrance diameters. For the exit diameter, a slight increasing trend can be observed with an increase in the scanning velocity, which may be attributed to the complicated material removal mechanism at the bottom of the transition hole. Since no assisted gas or liquid was employed in direct laser trepanning, hole formation is totally caused by the laser–material interaction, and realized gradually pulse by pulse. Each ultrashort ps laser pulse may result in melt expulsion, evaporation, or even phase explosion [30], whereas the removed material may partially deposit on the adjacent sidewall due to the limited space inside the forming micro-hole, leading to a reduced material removal rate compared with that on the open plane. It appears that a higher laser scanning speed may weaken the laser–material interaction at the transition hole bottom, leading to less material deposition and hence more material removal, so that an increased exit diameter may be expected. Considering the hole taper, a slight decreasing trend has also been noted, which corresponds to the varying trends of the entrance/exit diameters.

Figure 7e,f show the effects of the layer number and element number on the direct trepanning results, respectively, where marginally varied entrance diameters are noticed, and such trends can be explained in the same way as those related to the laser scanning speed. For the exit diameter, a slight increasing trend can be observed with an increase in the layer/element number, which may be attributed to the increased input laser energy. In addition, the slight declining trend regarding the hole taper can be depicted based on the varying trends of the entrance/exit diameters.

### 3.3. Effects of Parameters on Chemical-Assisted Laser Trepanning

For the chemical-assisted laser trepanning process, the measured results are summarized in Table 4, in which all of the parameter combinations yield through holes, as described in the last column. In addition, chemical-assisted laser trepanning is associated with wider holes both at the entrance and exit sides compared with the corresponding direct laser trepanning result, which may be mainly attributed to the serious mechanical effects caused by the liquid confined plasma and cavitation bubbles, as well as the strengthened chemical reaction between silicon and NaOH solution. Moreover, it appears that the standard deviation of the entrance diameter is generally less than that of the exit diameter, implying better roundness at the entrance than at the exit. This may be owing to the decrease of laser beam quality when penetrating the workpiece, as laser beam reflection at the sidewall, refraction at the liquid/plasma interface, and possible diffraction when interfering with the removed small particles may negatively affect the beam quality, resulting in unpredicted variance in the laser-material interaction at different positions along the laser scanning paths, and hence more obvious variance in the exit diameter. Using the chemical-assisted method, near damage-free through holes of about 260 μm in entrance diameter and around 2.36° in taper can be machined in the ~600 μm thick specimen.

**Figure 8:**
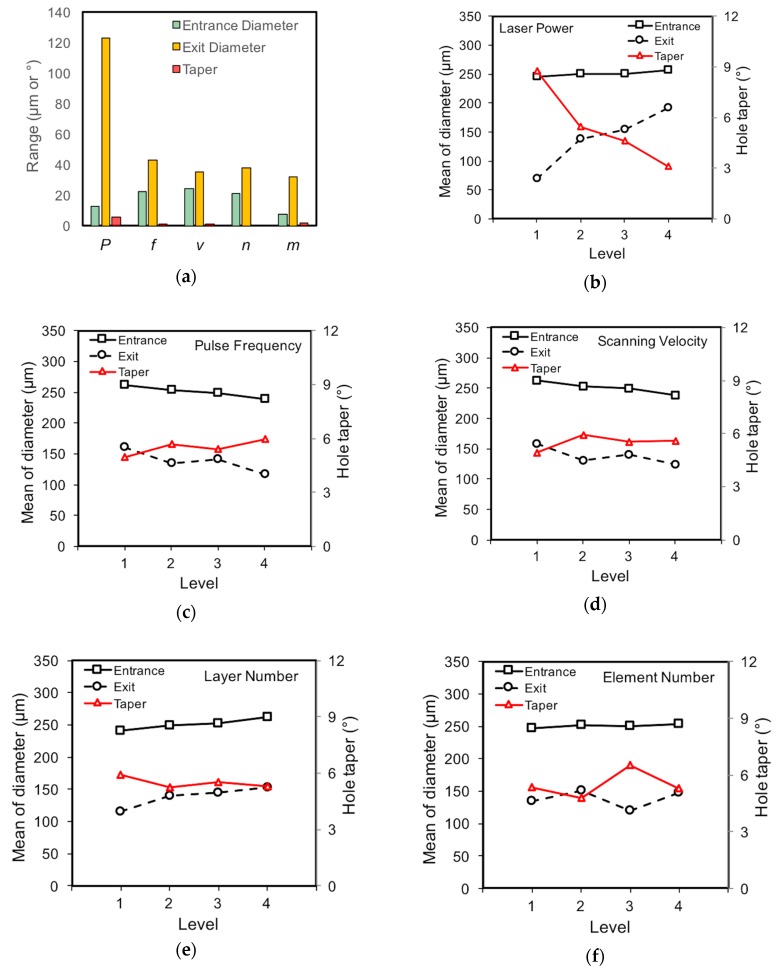
Effects of processing parameters on chemical-assisted trepanning results, where (**a**) is the range analysis result, and (**b**–**f**) correspond to the laser power, pulse frequency, scanning velocity, layer number, and element number, respectively.

Figure 8a illustrates the range value of the processing parameters with respect to the selected evaluation indicators of the hole profile, i.e., diameters at the entrance and exit, and hole taper. For the entrance diameter, the laser pulse frequency, laser scanning velocity, and layer number have comparably significant influences, followed by the laser power, while the element number has the smallest effect. Considering the exit diameter, the laser power is associated with the most significant influence and far surpasses the others, followed by the pulse frequency, while the rest of the parameters, i.e., laser scanning velocity, layer number, and element number, are of less significance. When the hole taper is discussed, again, the laser power has the most significant influence, while the effects of the other parameters are comparatively marginal.

The effects of laser power on the trepanning results are illustrated in Figure 8b, in which an increase in laser power from 11.5 to 32 W results in a slight increase in the entrance diameter and a rapid rise in the exit diameter. As a result, the steeper sidewall is expected, and hence the smaller hole taper. This may be explained from a view of laser energy loss after penetrating a liquid layer of a certain depth. In the assisted liquid, the transmitted laser intensity decreases with an increase in the liquid depth, and detailed calculation can be found in previous work [31]. It appears that the transmitted laser intensity arriving at the top surface is still high enough for material removal within the chosen laser power range in this study, and thus the entrance diameter varies slightly. In contrast, the transmitted laser energy reaching the transition hole bottom or the rear surface decreases comparatively significantly due to the severe interactions between the laser beam, plasma, and material in the limited hole space, where the incident laser power of a lower level yields a quite smaller exit diameter, while that of a higher level is still capable of producing a wider hole at the exit side.

Figure 8c depicts the effects of the laser pulse frequency on the hole diameter and taper, where an increase in the pulse repetition frequency results in a slight decrease both in the entrance and exit diameter, as well as a minor rise in the hole taper. Again, this can be attributed to the reduction of the single pulse energy caused by the higher laser frequency under the given laser power leading to less material removal per laser pulse, which effectively trades off the possible increasing trend of the material removal rate, owing to the accumulating effect of more engaging laser pulses, resulting in the aforementioned slight declining trends in hole diameters. Since the hole taper is estimated according to the diameters at the entrance and exit sides, a minor changing trend is therefore noticed.

Figure 8d shows the influence of laser scanning velocity on the trepanning result, where an increase in scanning speed is associated with a slight decrease both in entrance and exit diameter, as well as a marginal increase in hole taper. For higher scanning velocity, less input laser energy is deposited in a given position on the substrate, as the laser spot passes more quickly than in the lower scanning speed; therefore, less material removal is expected, and hence a reduced hole diameter. However, the varying trend regarding the exit diameter that is seen here seems inconsistent with the corresponding one in the direct laser trepanning case, as shown in Figure 7d. This variation may be attributed to the different situations regarding the ablated material expelling inside the transition hole. While the scanning speed-related deposition of ablated material on the adjacent sidewall may be obvious in the direct laser trepanning case and cause interference with the following laser pulses, such deposition can be effectively eliminated or minimized in the chemical-assisted process, owing to the enhanced shock wave of liquid-confined plasma and cavitation bubbles, and no deposited material is accumulated to affect the following material removal. Therefore, the laser energy arriving at the transition hole bottom/sidewall under different scanning speeds is fully used for removing material from the substrate during chemical-assisted machining, instead of reheating the thick recast layer, as in the direct laser machining case. As a result, the two machining methods are associated with different trends regarding the effects of the laser scanning speed on the exit diameter.

It is shown in Figure 8e that an increase in the layer number increases the entrance and exit diameters simultaneously, while the hole taper illustrates a slight decreasing trend. A larger layer number is associated with more feeding times and hence a smaller feeding step in the thickness direction; thus, a longer trepanning time and more input energy are expected, resulting in wider holes. However, such an increase in entrance diameter is not significant, which should be attributed to the selected layer number levels being generally sufficient to yield through holes, and more feeding times making a limited contribution to hole-widening. In a similar way, the increased element number results in a slight increase in the entrance diameter, as shown in Figure 8f, whereas the changing exit diameter and hole taper trends are slight and not monotonic, implying that the element number is the least important in affecting these two indicators.

Based on the above discussion, recommendations for the processing parameters in NaOH solution-assisted laser trepanning can be made. Since a steeper hole profile with a slightly increased entrance diameter and noticeably widened exit can be yielded by a larger laser power, a higher level (three or four) of laser power is preferred. For pulse frequency, a smaller level (one or two) is recommended, as the hole with a lower taper can be produced. When the laser-scanning velocity is considered, its influence on the hole formation is slight, and a higher level (three or four) is proper to employ from a view of machining efficiency improvement. Similarly, a lower level (one or two) of layer number and element number is suggested to reduce the trepanning time.

## 4. Discussion

### 4.1. Material Removal Mechanism in Direct Laser Trepanning Process

In the laser dry machining process, material removal is completely determined by the laser-material interaction, and is hence closely related to the pulse width of the incident laser. For a laser with a long pulse width, i.e., ns scale, a temperature field fully develops in the material via heat conduction after pulse energy absorption, leading to molten, evaporation, and even plasma formation. Therefore, material removal is caused by melt expulsion and evaporation, and the processing result is always associated with the heat-affected zone (HAZ), recast layer, spatters, and pile-ups near the structure edge [35,36]. In contrast, for an ultrashort laser, i.e., fs scale, material removal is realized through phase explosion before thermalization, and damage-free micro-structures can be achieved in a thermal way [37], so that thermal defects such as a HAZ, oxidation, and recast layer can be theoretically avoided. Considering the ps laser, its material removal mechanism is in a transition stage between the aforementioned ns laser and fs cases. Specifically, ps laser processing is capable of producing a micro-structure with a clear edge without an obvious HAZ under optimized parameters, while defects such as cracks, a recast layer, and edge fracture still can be found in many ps grooving results, as reported in previous work [30].

**Figure 9:**
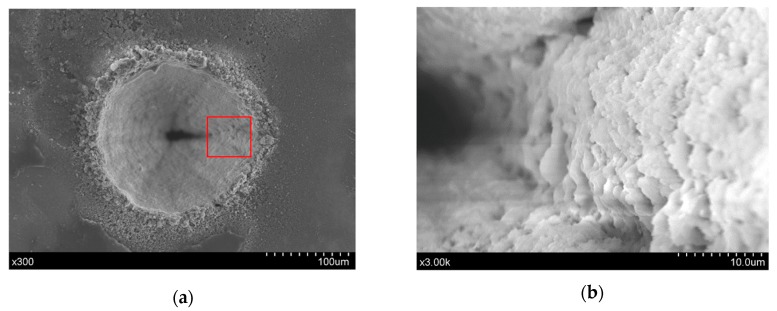
The laser dry trepanning results corresponding to test L4 in Table 3, where (**a**) is the whole view, while (**b**) depicts details of the irregular micro-humps distributed on the sidewall. The processing parameters include: *P* = 11.5 W, *f* = 1 MHz, *v* = 1200 mm/s, *n* = 25, and *m* = 120.

In this direct laser trepanning of silicon, material removal and hole formation are caused by the laser–material interaction, as no assisted liquid/gas is involved. Since the laser heating repeats at given times determined by the layer number, element number, and the laser heating paths, the accumulated thermal effects are significant, resulting in obvious thermal damage, such as the spatters and pile-ups surrounding the hole edge, as shown in Figure 4a. In addition, a dense distribution of irregular humps at the micron and sub-micron scale can be observed on the hole sidewall, as shown in Figure 9, which should be attributed to the incompleteness in material removal—especially when the hole is deep—and the following resolidification of the residual molten material.

**Figure 10:**
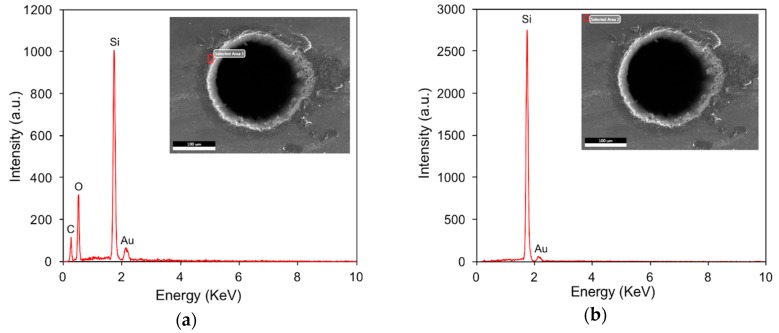
EDS spectra comparison at (**a**) hole edge and (**b**) non-ablated position. The red square in the upper-right figure represents the examined position. The process parameters include: *P* = 32 W, *f* = 0.4 MHz, *v* = 1200 mm/s, *n* = 15, and *m* = 100.

During direct laser trepanning, although the accumulated residual thermal effects are obvious, the high temperature field is still localized, which can be represented by the degree of surface oxidation. Figure 10 illustrates the comparison of EDS results between selected Area 1 next to the hole edge, and selected Area 2 with a distancet that is ~100 μm away from the trepanning position. Here, an evident O element has been detected in Area 1, as shown in Figure 10a, demonstrating the existence of an oxidation phenomenon. In contrast, the EDS results of Area 2 are free of O, indicating that the original material composition is maintained, which should be attributed to the location being about 100 μm away from the laser heating zone. Meanwhile, the oxidation phenomenon can be neglected, as the local temperature is marginally affected by the laser trepanning process.

**Figure 11:**
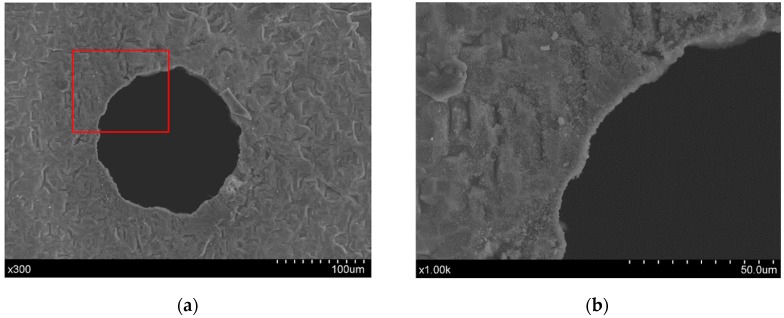
Exit details of direct micro-trepanning results, where (**a**) is an overall view, while (**b**) shows the details within the red square. The combination of parameters is the same as that in Figure 10.

At the exit side of the through hole trepanned by the direct laser method, the deposition of small particles/spatters can also be noticed, as shown in Figure 11. This further indicates that the through hole formation is a thermal-dynamic process caused by laser heating, as a severe laser-material interaction typified by explosion and spatter distribution on the surface can be noted both on the top and rear of the surface.

### 4.2. Material Removal Mechanism in Chemical-Assisted Laser Trepanning Process

**Figure 12:**
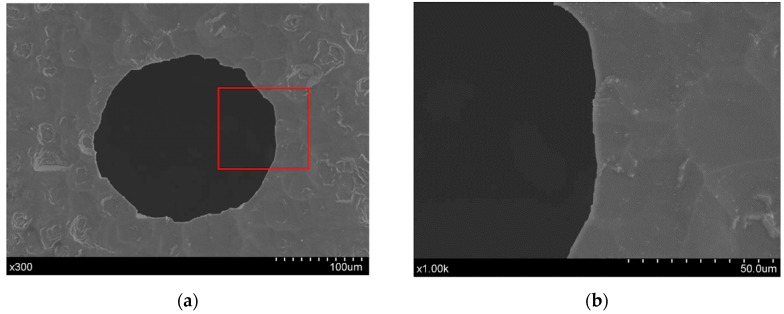
Exit details of the chemical-assisted micro-trepanning results, where (**a**) is an overall view, while (**b**) shows the details within the red square. The processing parameters include: *P* = 25.5 W, *f* = 0.8 MHz, *v* = 300 mm/s, *n* = 15, *m* = 120, and a NaOH solution with a concentration of one mol/L was used in chemical-assisted laser processing.

In NaOH solution-assisted laser trepanning, apart from the direct material removal caused by the interaction between the substrate and the transmitted laser beam, the laser-induced plasma and the following cavitation bubbles also play important roles in removing the material. The liquid-confined plasma zone behaves as a second heat source for silicon heating due to its high temperature property, while the expanding plasma zone and the subsequent cavitation bubbles result in serious mechanical effects on the substrate [32,38,39]. This leads to a larger heated zone and increased material removal rate, as well as the avoidance of particles deposition on the target surface or hole sidewall, so that wider holes with a cleaner surface are produced, as illustrated in Figure 4. In addition, such quality improvement can also be found in the rear surface, as illustrated in Figure 12, where a clean surface with significantly reduced particle deposition and sharp edges can be observed, demonstrating the advantage of chemical-assisted laser trepanning over direct laser processing.

In addition, the temperature field that is generated near the laser trepanning location enhances the chemical reaction between silicon and NaOH solution, as a higher local temperature increases the chemical etching rate [28], and therefore the material removal efficiency is accelerated. Further, the chemical redox reaction leads to material removal at the atomic or molecular level, which helps reduce the ablated surface roughness and hence improve the surface quality, as typified by the sidewall details shown in Figure 4b. Meanwhile, the chemical reaction not only exists within the short laser pulse duration, but also performs as a “post-processing” in pulse interval to further improve the surface quality, leading to a much smoother sidewall compared with that yielded by direct laser trepanning.

**Figure 13:**
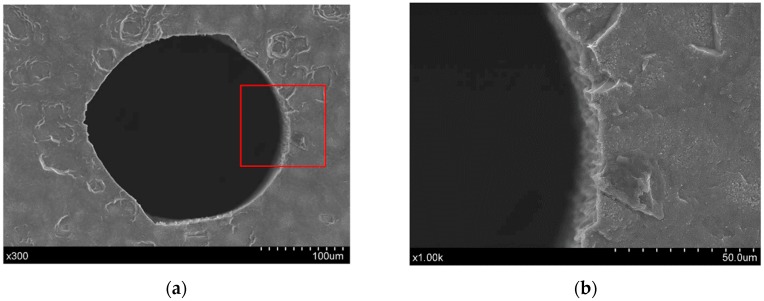
Exit details of the chemical-assisted micro-trepanning results, where (**a**) is an overall view, while (**b**) shows the details within the red square. The processing parameters include: *P* = 18.5 W, *f* = 0.4 MHz, *v* = 600 mm/s, *n* = 20, *m* = 120; and a NaOH solution with a concentration of one mol/L was used in the chemical-assisted laser processing.

However, in some parts of the hole sidewall, a quite coarse surface can also be noticed, as shown in Figure 13, which appears to be caused by the mechanical effect of the laser-induced plasma and the subsequent cavitation bubbles. Within laser pulse duration, dense free electrons can be produced via multi-photon ionization and impact ionization, leading to the formation of a plasma zone [40], which is of high temperature and high pressure, and exhibits an obvious shielding effect through absorbing the incident laser energy and a strong expelling effect via fast expanding in liquid media [32,38,39]. After a laser pulse, plasma decays rapidly and results in bubble generation in solution [40], which is always accompanied with shock wave and liquid jet impact, providing beneficial forces to remove the debris caused by laser machining. Meanwhile, the fast plasma collapse after laser pulse heating leads to a sudden drop in pressure, which likely results in the instantaneous expulsion of the vaporized and molten material [41,42]. These mechanical effects may roughen the ablated surface, resulting in the details that are shown in Figure 13b.

**Figure 14:**
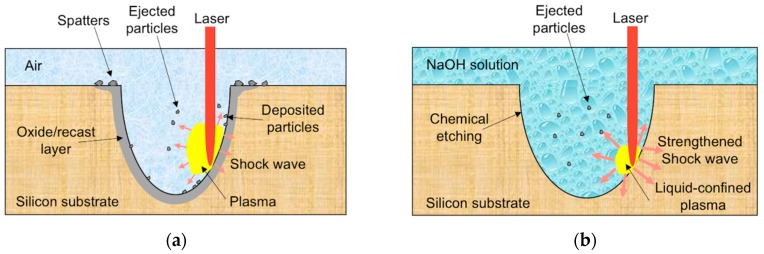
Schematic of material removal mechanism in (**a**) direct laser trepanning and (**b**) the chemical-assisted laser trepanning process.

The above analyses of material removal mechanisms can be summarized in Figure 14, where the scanning laser beam is heating a certain location of the transition hole sidewall. In a direct laser trepanning process carried out at atmosphere, local laser irradiation results in a temperature increase, phase change and plasma generation, followed by particle ejection and deposition, as well as the thermal damage formation that is typified by oxide and a recast layer, as shown in Figure 14a. In contrast, in the chemical-assisted case illustrated in Figure 14b, the laser-induced plasma is confined by the surrounding NaOH solution, leading to a higher inner pressure and hence strengthened shock wave on the substrate. In addition, the plasma zone degenerates into cavitation bubbles quickly after laser pulse heating, which then collapse and result in liquid jet impact and shock wave on the specimen. Therefore, material can be removed more efficiently and wider holes are yielded. Meanwhile, the ejected particle deposition can be eliminated or minimized, and the ablated material is flushed away effectively by the turbulent flow caused by the expansion and collapse of cavitation bubbles. Moreover, a chemical reaction between silicon and the assisted solution occurs at the liquid-solid interface, which further enhances the material removal rate and improves the surface quality.

## 5. Conclusions

An orthogonal experimental study of silicon trepanning has been conducted employing direct and chemical-assisted laser processing methods. Same experimental design was used, and the trepanning results were compared qualitatively via SEM/EDS observation and quantitatively through diameter measurement. Analyses of the effects of the parameters on the machining results were carried out, followed by an ample discussion of the material removal mechanisms. The main findings can be concluded as:

- Chemical-assisted laser trepanning is associated with obviously improved ablated surface quality in comparison to direct laser machining. Thermal defects have been observed in direct laser trepanning, including spatters on the top and rear surfaces, material oxidation near the hole entrance, and micro-scale and sub-micron scale humps on the rough sidewall. In contrast, it has been confirmed that better surface quality with a sharper hole edge, smoother sidewall, and neglected spatters area can be yielded by the chemical-assisted laser machining, which also results in no oxidation. In this study, near damage-free through micro-holes with small tapers down to 2.36° can be produced repeatedly using the NaOH solution-assisted laser trepanning method.
- Range analysis has been conducted. For laser dry trepanning, the pulse frequency outweighs the others in determining the entrance diameter, while the laser power and pulse frequency are the two parameters with more influence on the effects of the exit diameter and hole taper. In contrast, for NaOH solution-assisted laser machining, the laser power far surpasses the other parameters in affecting the exit diameter and hole taper, while the pulse frequency, scanning velocity, and layer number are associated with similar significance in affecting the entrance diameter. Based on the influence analysis, recommendations for the processing parameters have been given for chemical-assisted laser trepanning, including a higher laser power (level three or four), smaller pulse frequency (level one or two), faster laser scanning velocity (level three or four), and fewer layer numbers and element numbers (level one or two).
- Material removal mechanisms associated with the two trepanning methods have been amply discussed based on observations. The direct laser trepanning is accompanied with a thermal–dynamic material removal manner as the aforementioned thermal defects can be found, whereas material is removed in a more efficient way in the chemical-assisted case, as wider holes are yielded under the same processing parameters. The latter may be mainly attributed to the mechanical effects of the liquid-confined plasma and subsequent cavitation bubbles that enhance material removal. The smoother hole sidewall also highlights the benefits of the chemical reaction regarding minimizing surface roughness.

The performance evaluation and comparison that were carried out in this study demonstrate the advantages of the chemical-assisted ultrashort laser trepanning method over the direct laser processing method regarding the minimization of thermal defects, the improvement of surface quality, and the acceleration of ablation efficiency.

## Figures and Tables

**Table 1:** Material properties of single crystalline silicon [29].

Properties	Values
Density (Kg/m^3^)	2329
Heat capacity (J/(kg⋅K))	700
Thermal conductivity (W/(m⋅K))	130
Thermal diffusivity (m^2^/s)	7.974 × 10^−5^
Melting point (K)	1687

**Table 2:** The processing parameters used in this study.

Machining Parameters	Abbreviation	Levels			
		1	2	3	4
Laser power (W)	*P*	11.5	18.5	25.5	32
Pulse frequency (MHz)	*f*	0.4	0.6	0.8	1
Scanning velocity (mm/s)	*v*	300	600	900	1200
Layer number	*n*	10	15	20	25
Element number	*m*	60	80	100	120

**Table 3:** Summary of laser direct trepanning results.

Test No	*P*	*f*	*v*	*n*	*m*	Entrance Diameter (μm)	σ-Entrance (μm)	Exit Diameter (µm)	σ-Exit (µm)	Taper (°)	Note
L1	1	1	1	1	1	208.92	5.73	40.02	17.12	8.01	Through
L2	1	2	2	2	2	197.68	4.38	0.00	0.00	9.35	Blind
L3	1	3	3	3	3	181.12	5.05	0.00	0.00	8.58	Blind
L4	1	4	4	4	4	170.26	6.14	0.00	0.00	8.08	Blind
L5	2	1	2	3	4	206.87	5.10	126.60	6.09	3.83	Through
L6	2	2	1	4	3	180.57	6.88	48.01	9.49	6.30	Through
L7	2	3	4	1	2	177.64	8.26	0.00	0.00	8.42	Blind
L8	2	4	3	2	1	178.41	7.91	4.20	8.48	8.26	Through
L9	3	1	3	4	2	212.10	6.53	155.00	9.19	2.72	Through
L10	3	2	4	3	1	190.77	7.85	87.30	21.78	4.93	Through
L11	3	3	1	2	4	197.36	11.93	23.93	13.88	8.22	Through
L12	3	4	2	1	3	182.27	9.77	0.00	0.00	8.64	Blind
L13	4	1	4	2	3	200.88	8.48	160.37	3.17	1.93	Through
L14	4	2	3	1	4	203.40	7.43	110.94	13.45	4.41	Through
L15	4	3	2	4	1	197.23	7.73	92.20	10.36	5.00	Through
L16	4	4	1	3	2	169.28	13.18	28.28	18.79	6.70	Through

Note: σ-Entrance and σ-Exit represent the standard deviations of the entrance and exit diameters, respectively.

**Table 4:** Summary of chemical-assisted laser trepanning results.

Test No	*P*	*f*	*v*	*n*	*m*	Entrance Diameter (µm)	σ-Entrance (µm)	Exit Diameter (µm)	σ-Exit (µm)	Taper (°)	Note
L1	1	1	1	1	1	251.81	4.84	84.88	21.80	7.92	Through
L2	1	2	2	2	2	249.22	5.88	71.81	19.31	8.41	Through
L3	1	3	3	3	3	243.50	10.99	61.39	20.07	9.76	Through
L4	1	4	4	4	4	234.41	12.67	57.39	19.18	8.92	Through
L5	2	1	2	3	4	269.60	9.22	168.47	19.15	5.21	Through
L6	2	2	1	4	3	275.18	9.00	151.15	21.88	5.90	Through
L7	2	3	4	1	2	226.59	8.92	116.94	19.41	5.22	Through
L8	2	4	3	2	1	232.18	7.37	115.92	23.52	5.53	Through
L9	3	1	3	4	2	273.06	13.45	205.79	11.90	3.21	Through
L10	3	2	4	3	1	236.45	9.58	137.47	14.32	4.72	Through
L11	3	3	1	2	4	260.18	11.88	186.81	10.03	3.50	Through
L12	3	4	2	1	3	230.90	7.05	83.79	22.85	6.99	Through
L13	4	1	4	2	3	253.58	13.89	181.71	11.39	3.43	Through
L14	4	2	3	1	4	251.51	8.67	176.34	15.43	3.58	Through
L15	4	3	2	4	1	263.83	8.24	198.64	12.34	3.11	Through
L16	4	4	1	3	2	260.41	7.33	211.06	9.04	2.36	Through

Note: σ-Entrance and σ-Exit represent the standard deviations of the entrance and exit diameters, respectively.

## References

[1] Tan B. (2006). Deep micro hole drilling in a silicon substrate using multi-bursts of nanosecond UV laser pulses. J. Micromech. Microeng..

[2] Lee Y.H., Choi K.J. (2010). Analysis of silicon via hole drilling for wafer level chip stacking by UV laser. Int. J. Precis. Eng. Manuf..

[3] Tagliaferri F., Genna S., Leone C., Palumbo B., De Chiara G. (2017). Experimental study of fibre laser microdrilling of aerospace superalloy by trepanning technique. Int. J. Adv. Manuf. Technol..

[4] Pham K.X., Tanabe R., Ito Y. (2012). Trepanning drilling of microholes on cemented tungsten carbide using femtosecond laser pulses. J. Laser Appl..

[5] Ahn S., Hwang D.J., Park H.K., Grigoropoulos C.P. (2012). Femtosecond laser drilling of crystalline and multicrystalline silicon for advanced solar cell fabrication. Appl. Phys. A Mater. Sci. Process..

[6] Zhu H., Wang J., Yao P. (2016). A study of hybrid laser–waterjet micromachining of crystalline germanium. Proc. Inst. Mech. Eng. B J. Eng. Manuf..

[7] Wu Q., Wang J., Huang C. (2014). Analysis of the machining performance and surface integrity in laser milling of polycrystalline diamonds. Proc. Inst. Mech. Eng. B J. Eng. Manuf..

[8] Shi C., Ren N., Wang H., Xia K., Wang L. (2018). Effects of ultrasonic assistance on microhole drilling based on Nd:YAG laser trepanning. Opt. Laser Technol..

[9] Kaspar J., Luft A., Nolte S., Will M., Beyer E. (2006). Laser helical drilling of silicon wafers with ns to fs pulses: Scanning electron microscopy and transmission electron microscopy characterization of drilled through-holes. J. Laser Appl..

[10] Yilbas B.S., Akhtar S.S., Karatas C. (2011). Laser trepanning of a small diameter hole in titanium alloy: Temperature and stress fields. J. Mater. Process. Technol..

[11] Choudhury I.A., Chong W.C., Vahid G. (2012). Hole qualities in laser trepanning of polymeric materials. Opt. Laser Eng..

[12] Ashkenasi D., Kaszemeikat T., Mueller N., Dietrich R., Eichler H.J., Illing G. (2011). Laser trepanning for industrial applications. Phys. Procedia.

[13] Brandi F., Burdet N., Carzino R., Diaspro A. (2010). Very large spot size effect in nanosecond laser drilling efficiency of silicon. Opt. Express.

[14] Athanasiou C.-E., Bellouard Y. (2015). A monolithic micro-tensile tester for investigating silicon dioxide polymorph micromechanics, fabricated and operated using a femtosecond laser. Micromachines.

[15] Chanal M., Fedorov V.Y., Chambonneau M., Clady R., Tzortzakis S., Grojo D. (2017). Crossing the threshold of ultrafast laser writing in bulk silicon. Nat. Commun..

[16] Jiao L.S., Moon S.K., Ng E.Y.K., Zheng H.Y., Son H.S. (2014). Influence of substrate heating on hole geometry and spatter area in femtosecond laser drilling of silicon. Appl. Phys. Lett..

[17] Coyne E., Magee J.P., Mannion P., O’Connor G.M., Glynn T.J. (2005). STEM (scanning transmission electron microscopy) analysis of femtosecond laser pulse induced damage to bulk silicon. Appl. Phys. A Mater. Sci. Process..

[18] Jiao L.S., Ng E.Y.K., Wee L.M., Zheng H.Y. (2011). Role of volatile liquids in debris and hole taper angle reduction during femtosecond laser drilling of silicon. Appl. Phys. A Mater. Sci. Process..

[19] Li G., Hu S., Tang H., Chen B. (2018). Laser repeat drilling of alumina ceramics in static water. Int. J. Adv. Manuf. Technol..

[20] Wee L.M., Ng E.Y.K., Prathama A.H., Zheng H. (2011). Micro-machining of silicon wafer in air and under water. Opt. Laser Technol..

[21] Kaakkunen J.J.J., Silvennoinen M., Paivasaari K., Vahimaa P. (2011). Water-assisted femtosecond laser pulse ablation of high aspect ratio holes. Phys. Procedia.

[22] Nayak B.K., Gupta M.C., Kolasinski K.W. (2007). Ultrafast-laser-assisted chemical restructuring of silicon and germanium surfaces. Appl. Surf. Sci..

[23] Ehrlich D.J., Osgood R.M., Deutsch T.F. (1981). Laser chemical technique for rapid direct writing of surface relief in silicon. Appl. Phys. Lett..

[24] Kullmer R., Bäuerle D. (1987). Laser-induced chemical etching of silicon in chlorine atmosphere. Appl. Phys. A.

[25] Li L., Achara C. (2004). Chemical assisted laser machining for the minimisation of recast and heat affected zone. CIRP Ann..

[26] Hopman S., Fell A., Mayer K., Mesec M., Rodofili A., Kray D. (2009). Comparison of laser chemical processing and LaserMicrojet for structuring and cutting silicon substrates. Appl. Phys. A Mater. Sci. Process..

[27] Baumann S., Kray D., Mayer K., Eyer A., Willeke G.P. Comparative study of laser induced damage in silicon wafers. Proceedings of the 2006 IEEE 4th World Conference on Photovoltaic Energy Conference.

[28] Akhter P., Baig A., Mufti A. (1989). Dissolution of Si(100) layers in NaOH aqueous solutions. J. Phys. D Appl. Phys..

[29] Tangwarodomnukun V., Wang J., Huang C.Z., Zhu H.T. (2012). An investigation of hybrid laser-waterjet ablation of silicon substrates. Int. J. Mach. Tool. Manuf..

[30] Zhu H., Zhang Z., Xu J., Xu K., Ren Y. (2018). An experimental study of micro-machining of hydroxyapatite using an ultrashort picosecond laser. Precis. Eng..

[31] Zhu H., Wang J., Yao P., Huang C.Z. (2017). Heat transfer and material ablation in hybrid laser-waterjet microgrooving of single crystalline germanium. Int. J. Mach. Tool. Manuf..

[32] Wu B. (2008). High-intensity nanosecond-pulsed laser-induced plasma in air, water, and vacuum: A comparative study of the early-stage evolution using a physics-based predictive model. Appl. Phys. Lett..

[33] Wu B., Shin Y.C. (2005). A self-closed thermal model for laser shock peening under the water confinement regime configuration and comparisons to experiments. J. Appl. Phys..

[34] Zhu X., Naumov A.Y., Villeneuve D.M., Corkum P.B. (1999). Influence of laser parameters and material properties on micro drilling with femtosecond laser pulses. Appl. Phys. A Mater. Sci. Process..

[35] Ki H., Mohanty P.S., Mazumder J. (2001). Modelling of high-density laser-material interaction using fast level set method. J. Phys. D Appl. Phys..

[36] Von der Linde D., Sokolowski-Tinten K. (2000). The physical mechanisms of short-pulse laser ablation. Appl. Surf. Sci..

[37] Chichkov B.N., Momma C., Nolte S., von Alvensleben F., Tunnermann A. (1996). Femtosecond, picosecond and nanosecond laser ablation of solids. Appl. Phys. A Mater. Sci. Process..

[38] Peyre P., Berthe L., Fabbro R., Sollier A. (2000). Experimental determination by PVDF and EMV techniques of shock amplitudes induced by 0.6-3 ns laser pulses in a confined regime with water. J. Phys. D Appl. Phys..

[39] Hammer D.X., Jansen E.D., Frenz M., Noojin G.D., Thomas R.J., Noack J., Vogel A., Rockwell B.A., Welch A.J. (1997). Shielding properties of laser-induced breakdown in water for pulse durations from 5 ns to 125 fs. Appl. Opt..

[40] Noack J., Vogel A. (1999). Laser-induced plasma formation in water at nanosecond to femtosecond time scales: calculation of thresholds, absorption coefficients, and energy density. IEEE J. Quantum Electron..

[41] Saxena I., Ehmann K., Cao J. (2015). High throughput microfabrication using laser induced plasma in saline aqueous medium. J. Mater. Process. Technol..

[42] Pallav K., Han P., Ramkumar J., Nagahanumaiah, Ehmann K.F. (2013). Comparative assessment of the laser induced plasma micromachining and the micro-EDM processes. J. Manuf. Sci. Eng..

